# E-MPATHY: Development, validation, reliability, and acceptability of a decision-support tool for district-level maternal death review in Indonesia

**DOI:** 10.1186/s12884-026-09735-5

**Published:** 2026-07-30

**Authors:** Ratnasari Dwi Cahyanti, Widyawati Widyawati, Mohammad Hakimi

**Affiliations:** 1 Obstetrics and Gynecology Department, Faculty of Medicine, Jl. Prof. Soedarto, Universitas Diponegoro, Tembalang, Semarang, Jawa Tengah 50275 Indonesia; 2 Doctoral Program, Faculty of Medicine, Public Health and Nursing, Universitas Gadjah Mada, Yogyakarta, Indonesia; 3 Pediatric and Maternity Nursing Department, Faculty of Medicine, Public Health and Nursing, Universitas Gadjah Mada, Yogyakarta, Indonesia; 4 Obstetrics and Gynecology Department, Faculty of Medicine, Public Health and Nursing, Universitas Gadjah Mada, Yogyakarta, Indonesia

**Keywords:** Maternal death review, Decision-support tool, Scientific evidence, Clinical standard, Three delays model, Evidence-based recommendation, District levels, Learning environment, Maternal death surveillance and response, Indonesia

## Abstract

**Background:**

Maternal Death Review (MDR), the cornerstone of Maternal Death Surveillance and Response (MDSR), generates evidence to inform strategies for reducing maternal death. However, MDR is often constrained by blaming cultures and the lack of structured approaches for systematic case review. These limitations hinder the objectivity identification of causes, contributing factors, and actionable recommendations. To address this gap, we developed a novel structured decision-support tool (DST) integrating scientific evidence and clinical standards to formulate evidence-based recommendation. This study aimed to develop a DST for district-level MDR and to evaluate its validity, reliability, and acceptability.

**Methods:**

The tool development comprised four steps: needs assessment, face and content validity, reliability, and acceptability assessment. We compared the review findings of 113 cases based on expert-decision constructed using a DST between district and provincial review teams in Central Java, Indonesia. Reviewers’ acceptability was assessed through observations during the review and analyzed using the Theoretical Framework of Acceptability.

**Results:**

The needs assessment resulted in 1) naming the tool (E-MPATHY), reflecting a supportive learning environment, and 2) the development of a structured DST framework integrating scientific evidence with the three delays model across pregnancy periods. The tool guided reviewers in assigning underlying causes of death based on the International Classification of Diseases for Maternal Mortality (ICD-MM), assessing preventability, identifying contributing factors, and generating recommendations. Agreement between two reviewer teams was almost perfect for ICD-MM classification (κ = 0.86; 95%CI:0.76–0.96;* p* < 0.001). In the three delays assessment, the highest agreement observed in the postpartum period (κ = 0.58; 95%CI:0.42–0.74; *p* < 0.001), with substantial agreement in the third delay phase (κ = 0.61; 95%CI:0.46–0.75; *p* < 0.001). The reviewers’ acceptability indicated the benefits of evidence-based review and encourages positive learning attitudes in their daily practices.

**Conclusion:**

The E-MPATHY had almost perfect inter-rater reliability in assigning ICD-MM. In addition, there was convincing evidence of its validity and acceptability to be a support direct learning tool potentially applied to institutionalized workable district MDR in Indonesia. However, as the evaluation was conducted within one province, further studies are needed to assess the tool’s applicability across diverse health system contexts.

**Supplementary Information:**

The online version contains supplementary material available at https://doi.org/10.1186/s12884-026-09735-5.

## Background

The third Sustainable Development Goal (SDG-3) calls for reducing the global maternal mortality ratio to less than 70 per 100,000 live births by 2030. However, maternal mortality remains a major global health challenge, with an estimated 712 women dying per day from preventable complications related to pregnancy and childbirth in 2023 [1, 2] Despite the excess availability of quality evidence to inform policymaking and clinical practice, implementing effective interventions to prevent maternal death remain challenging [3–5].

To address this issue, the World Health Organization (WHO) introduced the Maternal Death Surveillance and Response (MDSR) system in 2013 as a systemic approach to strengthening the monitoring and prevention of maternal deaths. The MDSR framework follows a continous action process that mirrors a typical audit cycle, consisting of identification and notification of cases, maternal death review (MDR), analysis and recommendation, and response and monitoring. As its core component, MDR provides a structured analysis of the underlying causes and contributing factors of maternal deaths to inform effective preventive action [6, 7].

High-quality MDR requires reliable evidence, clear standards, and structured review processes [6, 7]. To support standardized classification, the WHO introduced the International Classification of Diseases for Maternal Mortality (ICD-MM), derived from the Tenth Revision of International Classification of Diseases (ICD-10). This classification system has been widely adopted to improve consistency in defining maternal deaths and identifying their causes [8].

However, classification, while essential, is insufficient to support comprehensive MDR processes. Evidence from our previous systematic review and meta-analysis shows that existing MDR tools largely emphasize assigning causes of death using ICD-MM, with limited capacity to support deeper analysis, patterns identification, and evidence-informed decision-making [9]. In particular, the absence of explicit clinical standards and structured guidance may lead to inconsistent cause of death and hinder the identification of root causes, preventable factors, and patterns of inappropriate care across reviewers, potentially leading to bias [6, 10].

In addition, MDR quality depends on reliable data, skilled reviewers, and a supportive learning environment [11–13]. Several studies have highlighted persistent barriers to obtaining complete and accurate data, often driven by an unfavorable institutional “blame culture”. This culture creates a vicious cycle of reluctance to disclose critical information, subjectivity in case analysis, and poor feedback for quality improvement [14, 15]. It may also discourage open identification of care deficiencies, particularly across hierarchical levels of care, reinforcing punitive perceptions rather than learing-oriented review processes. As a result, MDR processes may fail to generate actionable, evidence-based insights to improve maternal health outcomes [16].

To address these gaps, we developed a decision-support tool (DST) to facilitate MDR processes in generating structured, evidence-based recommendations within a supportive learning environment. The development of the tool was guided by Armenakis and Bedeian’s theory of organizational change, as translated by Pattinson and Berg, which emphasizes both the content of change (causes of maternal death and recommended actions) and the process of implementation [17, 18]. Evidence suggests that addressing the content of change at the district level is particularly important, as contributing factors may vary across setting even for similar causes of death, leading to different prevention strategies [13]. This need is particularly evident in the Indonesian context, where district-level maternal death reviews have been conducted as part of the national Maternal and Perinatal Audit Surveillance and Response (AMP-SR) program [19]. Strengthening MDR at this level therefore requires tools that can systematically support context-specific analysis and recommendation development. Accordingly, this study aimed to develop and evaluate a decision-support tool for district-level MDR, with a focus on assessing its validity, reliability, and acceptability.

## Methods

The decision-support tool was developed with multiphase sequential mixed-methods (qualitative– quantitative—[quantitative + qualitative]) [20]. Tool development comprised four steps: need assessment, face and content validity, reliability, and acceptability assessment. This study was conducted in Central Java Province, Indonesia, which contributes to around 15% of Indonesia’s population and is one of the provinces with a number of maternal mortality rate (MMR) of 110.41/100,000 live-births [21].

### Need assessment

Focus group discussions (FGDs) were purposively conducted in five districts in Central Java, selected to represent a diverse range of geographical conditions, maternal death numbers, maternal mortality rates (MMRs), types of maternal mortality cases, educational qualifications of the maternal death audit (MDA) committee and the activeness of the district health office (DHO) in conducting the audit cycle. In each FGD, the informants were 7–8 members of the MDA district committee. The committee consists of a management team (DHO) and internal reviewers, the facility-based professionals and regional representatives of professional association.

The conceptual framework of the content of change in the Confidential Enquiry into Maternal Deaths (CEMD) developed by Pattinson et al. [18] was used to conduct the thematic data analysis consisting of two domains of concepts/needs (cause of death and recommendations) (see Appendix 1). A panel of seven obstetrics and gynecology (OB/GYN) licensed practitioners with expertise in maternal health programs, representing different regions of Indonesia (western, central, and eastern areas), was convened to evaluate the thematic content and feasibility of the proposed concepts, indicators, and items. Each expert independently assessed the proposed indicators using a Likert scale questionnaire, rating their relevance to maternal death review and feasibility for implementation in practice. The scoring results were summarized and discussed in a panel meeting to reach consensus (see Appendix 2).

The name of “audit” in Indonesia, is commonly used to represent the MDR and the action cycle of the maternal audit itself. One of the panel’s consensuses was concerned with ameliorating the word “audit”, which had a connotation of inspection [22] and not supporting the learning environment. The term “empathy” was proposed, defined as the action of understanding, being aware of, being sensitive to, and vicariously experiencing the feelings, thoughts, and experience of another [23]. This term reiterates the need to internalize the basic principles of the maternal audit as an effort to prevent similar maternal death and to avoid the contra-productive actions. The tool was named 'E-MPATHY' (**E**lectronic-I**mp**lementation of M**a**ternal Dea**th**/Mortalit**y** Review), with the initial letter separated by a dash to represent the development of the electronic-based decision-support tool. Additionally, the panel members agreed to utilize the scientific evidence not only to identify deficiencies in maternal care but also to offer recommendations.

In the developing of recommendations for maternal death review, the three delays model was proposed as a conceptual framework of a solution-based approach in each pregnancy period: identification of the decision to seek care, ability to reach care, and ability to receive adequate care, respectively [24]. The three delays model was used to assign the preventive strategies, not only in identifying delays when complication occurs [25]. The identified contributing factors were categorized based on its preventability for appropriate recommendations with specific target (community, health facilities, professional organization, DHO, and local government). The consensus also highlighted the need to use a framework to efficiently assess the delays in each pregnancy period (antenatal, early pregnancy, ante-intrapartum and postpartum periods) based on clinical guidelines.

### Tool development and validity

The components of the E-MPATHY Draft I were developed in the website: e-mpati.net, according to the findings of the needs assessment transformed into the three MDR forms (community autopsy tool for maternal death, a summary form for maternal death in facilities, and narrative summaries of a committee worksheet). The access for the specific MDR form is restricted to a specific user’s role (health facilities/DHO/reviewers) for confidentiality (see Appendix 3).

Five licensed OB/GYN practitioners with extensive professional experience in maternal health programs, representing western, central, and eastern Indonesia, assessed content validity. In the validation stage, the item content validity index (I-CVI) and scale content validity index (S-CVI) were determined using Lynn's framework [26]. For five experts, an I-CVI of 1.00 was considered acceptable. The I-CVI was calculated based on experts rating using a three-point scale (0 = irrelevant or very irrelevant, 1 = not relevant, and 2 = relevant). To assess the overall scale validity, the S-CVI (universal agreement) was calculated as the proportion of items rated as relevant by all experts, with a minimum acceptable value of 0.8. All items achieved an I-CVI of 1.00, indicating universal agreement among experts. Minor clarifications were confirmed during a brief panel discussion, and no substantial revisions were required.

Face validity of E-MPATHY was assessed using feedback from 10 members of the MDA committees at Banyumas and Brebes districts. The tool's display, readability, and usability were assessed by a questionnaire during the validation process. Numerical scores were assigned by the research team after participants answered the survey questions using a 5-point Likert scale (1 = extremely unclear, 2 = unclear, 3 = fairly clear, 4 = clear, and 5 = extremely clear). At the validation phase, some items were removed or added resulting in E-MPATHY Draft II.

### Pilot and reliability

District- and provincial-level reviewers were involved in piloting the decision-support tool and assessing inter-rater reliability. District reviewers consisted of two hospital-based general obstetrician and gynecologists, from the Pekalongan residency. Provincial reviewers were appointed by the Indonesian Society of Obstetrics and Gynecology (POGI) as its representatives in Central Java, consisting of one expert in maternal–fetal medicine and one expert in social obstetrics and gynecology. All the reviewers were members of the team responsible for conducting the facility-based MDR.

A pilot test was conducted to identify potential issues related to tool use during MDR at district level. Prior to the pilot test and reliability assessment, all reviewers received structured training on tool use and standardized assessment of direct and indirect causes of maternal deaths. Feedback from the pilot test led to several improvements. A navigation bar was added to facilitate case selection and to display key components, including summary forms for maternal deaths in facilities, scientific evidence, and systematic analysis across each pregnancy period. Based on the findings of the pilot test, the final version of E-MPATHY was developed and subsequently subjected to reliability testing.

Secondary data obtained from completed verbal autopsy reports and maternal death summary forms from health facilities in Central Java over five consecutive years were used. The forms were scrutinized and transformed into the E-MPATHY format by chronologically categorizing the data into different pregnancy periods by research assistants under the supervision of the principal investigator. Records with missing information on key variables required to categorize deaths across pregnancy periods were excluded from the analysis (complete case analysis).

**Fig. 1:**
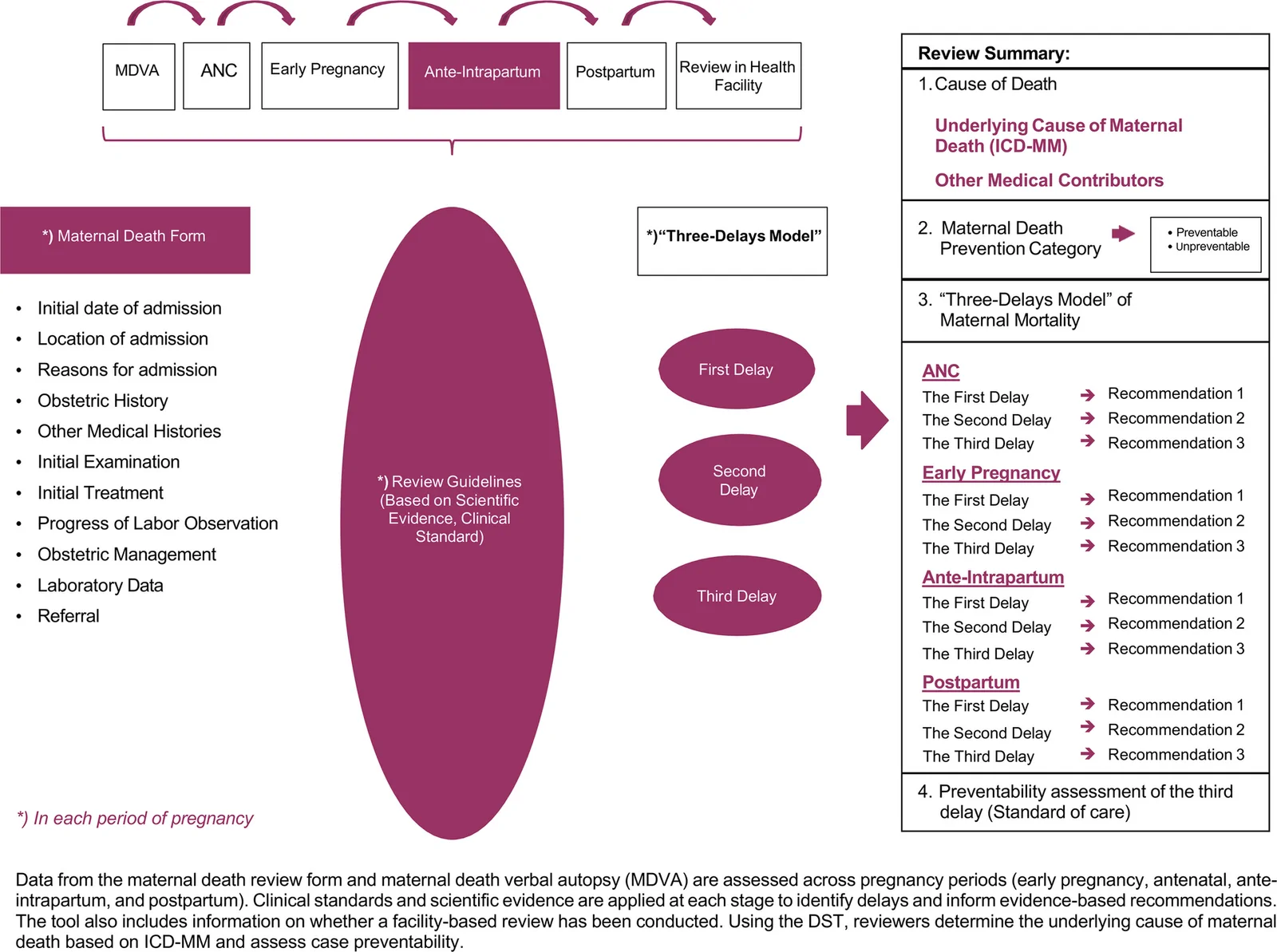
Flow Framework of E-MPATHY

A total of 113 maternal deaths were reviewed to measure inter-rater reliability conducted by two groups (district and provincial reviewers). The reviews were conducted using the Zoom platform. Reviewers’ consensus determinations were presented on a structured form (reviewer worksheet) in the tool. The consensus determination analyzed the underlying causes of death using the ICD-MM, category of potential preventability cases, contributing factors of maternal deaths (the three delays), and generated recommendations of the specific target outcomes (Fig. 1). Inter-rater reliability was evaluated with the weighted Kappa statistics of nominal and ordered categorical variables. Bootstrap estimation of standard error for Kappa was applied to calculate 95% confidence intervals (CI). The Landis and Koch classification of inter-rater reliability was used to interpret the coefficient [27]. The Kappa statistic (strength of agreement) was classified based on Landis and Koch criteria: poor (κ < 0.00), slight (κ = 0.00–0.20), fair (κ = 0.21–0.40), moderate (κ = 0.41–0.60), substantial (κ = 0.61–0.80), and almost perfect (κ = 0.81–1.00). The SPSS version 25 (IBM Corp., Armonk, NY, USA) was used to analyze Fleiss kappa values with 95% CI.

### Acceptability

A qualitative study using observation methodology was conducted to access the reviewer’s acceptability of E-MPATHY in formulate evidence-based recommendation during MDR. Data were collected during the reliability testing via Zoom by RC. Observations were recorded in field notes taken using a prestructured form guided by the Theoretical Framework of Acceptability (TFA) [28] detailing the time and setting, a brief description of the situational context and participants. We reflected on the observation process with other researchers (MH and WW). A deductive thematic analysis was conducted using the seven TFA constructs (Affective Attitude; Burden; Ethicality; Intervention Coherence; Opportunity Costs; Perceived Effectiveness; and Self-efficacy) [28] as a predefined coding framework. Observational data were coded and categorized according to these constructs, without modification or addition of new constructs. During the analysis, we used memos to clarify codes and to record ideas and impressions.

### Ethical considerations

The study protocol complied with the 7 (seven) WHO 2011 ethics standards. The study received ethical approval No. 102/EC/KEPK/FK UNDIP/2019 from the Research Ethics Committee, Faculty of Medicine, Universitas Diponegoro, Semarang, Central Java. Permission and approval were obtained from the Government of Central Java and Brebes, Pekalongan, Kendal, Semarang, Kudus, Banyumas District Health Offices, Central Java. Prior to the data collection, all participants provided written informed consent for audio-recording and using excerpts in and reports and publication. The participants remained anonymous during the analysis and presentation of findings.

## Results

### Validation

Validation of the E-MPATHY Draft I demonstrated good content and face validity (display, readability, and usability) were assessed by a questionnaire during the validation process. The I-CVI was 1.00 from all items in introduction, verbal autopsy, form for maternal death review in facilities, clinical standards, reviews, narrative summaries of a committee worksheet, and plan of action. The S-CVIs for all items indicated an excellent content validity (S-CVI = 1.00).

### Inter-rater reliability

One-hundred-thirteen (113) maternal deaths were reviewed and included in the inter-rater reliability analysis between the district and provincial reviewers using E-MPATHY. Using the ICD-MM, the most common underlying causes of death were considered. Table 1 compares the ICD-MM underlying cause groups and summarizes levels of agreement between the two MDR committees. The overall kappa (κ) was 0.86 (95% CI: 0.76–0.96); *p* < 0.001), with the highest agreement for abortive outcomes (κ = 1), obstetric hemorrhage (κ = 0.96), and non-obstetric complications (κ = 0.82). The lowest agreement was for pregnancy-related infection and other obstetric complications with κ = 0.71.

**Table 1:** Level of agreement of ICD-MM group classification for underlying causes of death by district and provincial MDR committees

ICD-MM Group number	ICD-MM group name	District team n (%)	Provincial team n (%)	Kappa (95% CI)^a^	Kappa (95% CI)^b^
1	Pregnancy with abortive outcome	2 (1.8)	2 (1.8)	1.00 (0.87–1.00)	
2	Hypertensive disorders in pregnancy, childbirth and the puerperium	31 (27.4)	31 (27.4)	0.82 (0.64–1.00)	
3	Obstetric hemorrhage	41 (36.3)	41 (36.3)	0.96 (0.78–1.00)	
4	Pregnancy-related infection	5 (4.4)	6 (5.3)	0.71 (0.53–0.89)	
5	Other obstetric complications	6 (5.3)	5 (4.4)	0.71 (0.53–0.89)	0.86 (0.76–0.96)
6	Unanticipated complications of management	0	2 (1.8)	n/a	
7	Non-obstetric complications	18 (15.9)	20 (17.7)	0.94 (0.75–1.00)	
8	Unknown/undetermined causes of death	10 (8.8)	6 (5.3)	0.73 (0.55–0.91)	
9	Coincidental causes	0	0	n/a	

*n/a* not accounted, a, Inter-raters’ Fleiss kappa for individual categories; b, Inter-raters’ Fleiss kappa for overall; CI, confidence interval; ICD-MM, International Classification of Diseases-Maternal Mortality; MDR, maternal death review*.*

The district committee identified 86 of the 113 cases (76.1%) were preventable, whereas the provincial MDR committees assessed 97 (85.8%) as preventable. Overall, there was substantial agreement between preventable events assigned by the district and provincial MDR committees (κ = 0.68, 95% CI: 0.50–0.87; *p* < 0.001).

Furthermore, to determine the underlying preventable factors, the three delays model was applied to identify the delays in each pregnancy period (Table 2). The provincial reviewers identified more delays in all three categories than the district reviewers. The highest agreement in identification of delays in facilities was found in the third phase delay of the postpartum period (κ = 0.61, 95%CI: 0.46–0.75;* p* < 0.001). The ante-intrapartum period had the lowest agreement (κ = 0.21, 95%CI: 0.05–0.36; *p* < 0.001).

**Table 2:** Level of agreement between district and provincial MDR committees in identifying the three delays across pregnancy period

	**District team** **n (%)**	**Provincial team** **n (%)**	**Kappa (95% CI)**
**Antenatal**	**57 (50.4)**	**80 (70.8)**	**0.45 (0.33–0.57)**^**b**^
Phase one delay	27 (23.9)	26 (23.0)	0.63 (0.46–0.80)^a^
Phase two delay	0	0	n/a
Phase three delay	40 (35.4)	78 (69.0)	0.39 (0.27–0.52)^a^
**Early pregnancy**	**4 (3.5)**	**8 (7.1)**	**0.48 (0.33–0.62)**^**b**^
Phase one delay	3 (2.7)	1 (0.9)	0.49 (0.11–1.00)^a^
Phase two delay	1 (0.9)	0	n/a
Phase three delay	1 (0.9)	7 (6.2)	0.24 (0.15–0.66)^a^
**Ante-intrapartum**	**47 (41.6)**	**79 (69.9)**	**0.21 (0.05–0.36)**^**b**^
Phase one delay	12 (10.6)	5 (4.4)	0.31 (0.01–0.61)^a^
Phase two delay	0	0	n/a
Phase three delay	41 (36.3)	77 (68.1)	0.29 (0.16–0.43)^a^
**Postpartum**	**54 (47.8)**	**58 (51.3)**	**0.58 (0.42–0.74)**^**b**^
Phase one delay	10 (8.8)	3 (2.7)	0.28 (0.04–0.60)^a^
Phase two delay	0	0	n/a
Phase three delay	48 (42.5)	54 (47.8)	0.61 (0.46–0.75)^a^

*n/a* not accounted, a, Inter-raters’ Fleiss kappa for individual categories; b, Inter-raters’ Fleiss kappa for overall. *CI* Confidence interval, *MDR* Maternal death review

Table 3 presents an example of reviewers’ recommendations derived from a maternal death case review. The example illustrates how E-MPATHY was used to identify underlying factors contributing to delays across the antenatal, intrapartum, and postpartum periods and to formulate recommendations tailored to the specific findings of the case. The recommendations and their targets were determined by the review team based on the identified contributing factors and health system gaps observed during the review process. These findings included barriers related to care-seeking, access to health services, and the provision of care across the health facilities involved. The case was classified as ICD-MM Group 2 (hypertensive disorders in pregnancy, childbirth, and the puerperium) and assessed as preventable, with contributing conditions including obesity, advanced maternal age, and anemia. Based on the identified delays across the antenatal, intrapartum, and postpartum periods, the recommendations focused on improving early recognition of danger sings at the community level, strengthening clinical management and referral systems at health facilities, and enhancing professional support through feedback and continuing medical education. These efforts were supported by adherence to clinical standards, regular monitoring and evaluation of service quality, and the implementation of emergency drills. The resulting recommendations targeted multiple levels of the health system, including the community, health facilities, district health authorities, and professional organizations (IBI, IDI, and POGI), according to the contributing factors identified during the review.

**Table 3:** Illustrative examples of the recommendations formulated by the maternal death review team using E-MPATHY decision-support tool

Underlying cause of maternal death Category			2 Hypertensive disorders in pregnancy, childbirth, and the puerperium O15.2 Eclampsia in the puerperium		
Contributing condition			E66 Obesity O09.519 Advanced maternal age O99.0 Anaemia complicating pregnancy, childbirth and the puerperium		
Preventability			Preventable Substandard care		
Component	Delays	Target	Recommendation	Person in Charge	Scheduled time
Antenatal	First delay Low awareness and poor recognition of danger signs, lack of antenatal visit	Community	Training and mobilization of health cadres to support pregnant women with high-risk pregnancies	Head of Community Health Center, DHO	Three months
			Health education involving religious leaders (mosque-based maternal health promotion)	DHO, Village Head, Department of Religion (at regional level)	One month
	Third delay Early detection: Health professional: misdiagnosis, inadequate management of follow-up care	Community health center	Quality improvement focus: Preeclampsia risk assessment, monitoring, and referral management. Approach: integrated ANC with supervision and periodic training (every 3 months)	Head of Community Health Center, District MCH coordinator	Three months
		Professional organization	Feedback from health professional organization to its members	POGI	One month
		Hospital, Community health center	Continuing medical education aligned with MDR recommendations on current preventive measures at facility and community levels	DHO	Three months
Intrapartum	Third delay Standard management: Labor monitoring, Diagnosis, Stabilization	Professional organization	Feedback from health professional organization to its members	POGI	One month
			Continuing medical education aligned with MDR recommendations on current preventive measures	POGI, IDI, IBI	Three months
		Hospital	Regular emergency drills (per 3 months)	Hospital Director	One month
Postpartum	Third delay Standard management: Postpartum monitoring: Early detection, diagnosis Emergency condition: Early detection, diagnosis, stabilization	Hospital	Review of clinical standards for postpartum management of eclampsia	Hospital Director	One month
			Monitoring and evaluating quality of care indicators through monthly EmOC meetings		
			Regular emergency drills (per 3 months)		

Second-delay factors were assessed, but none were identified in this illustrative case

*ANC* Antenatal care, *EmOC* Emergency obstetric care, *IDI* *Ikatan Dokter Indonesia* (Indonesian Medical Association), *MCH* Mother/child health, *MDR* Maternal death review, *POGI* *Perkumpulan Obstetri dan Ginekologi Indonesia* (Indonesian Society of Obstetrics & Gynecology), *Ikatan Bidan Indonesia* (Indonesian Midwives Association)

Across all reviewed cases, the recommendations formulated by the maternal death review teams with support from the E-MPATHY varied considerably in content and scope and were spesific to the findings identified during each review. As a result, Fleiss’ κ statistics were not calculated to assess interrater agreement for the recommendation component.

### Acceptability

Table 4 displays the key themes and sub-themes generated from the TFA analysis reflecting participants’ concurrent acceptability of E-MPATHY for decision support tool in MDR.

**Table 4:** Assessments of the seven dimensions of acceptability

Key Themes	Affective Attitude	Burden	Ethicality	Intervention coherence	Opportunity Costs	Perceived Effectiveness	Self-efficacy
**Representing each TFA construct**	**How an individual feels about the intervention**	**The perceived amount of effort that is required to participate in the intervention**	**The extent to which the intervention has a good fit with an individual's value system**	**The extent to which a participant understands the intervention**	**The extent to which benefits, profits, or value must be given up to engage in the intervention**	**The extent to which the intervention is perceived as likely to achieve its goal**	**The participants’ confidence that they can perform the behavior(s) required to participate in the intervention**
E-MPATHY tool	Perceived benefits of evidence-based decision-making in review	The review model encouraged reviewers to update their knowledge of the latest contents in the maternal and child health handbook	Perceive behavioral changes in daily practices	Reviewers constantly used clinical guidelines to resolve disagreement	Using E-MPATHY in reviews led to more appropriate alternative recommendations	The first experience of adapting reviews using clinical guidelines to assign cause of death and identify delays	Reviewers were able to routinely review more cases per day
	Suboptimal clinical management led to fatal outcomes	Reviewers were given time to learn updated clinical guidelines	Self-reflection following the identification of the third delay	Reviewers’ view on E-MPATHY as an easy-to-use tool for facilitating the review process	Perceived learning and updating reviewers’ knowledge	Perceived difficulties in initiating the search for new guidelines for non-obstetric cases	
			Changes in reviewers’ attitudes to prevent delays in intervention at referral hospitals	Desire to use the E-MPATHY for reviews at the district level	Reviews were more concise and accurate with the use of guidelines	E-MPATHY enables reviewers to resolve disagreements through literature searches when guidelines were unavailable	
					Minimal mis-classification of the cause of maternal death	Variation in assessing preventability amid frequent clinical updates, task-shifting, and indirect delays due to inadequate laboratory standard	

*E-MPATHY* Electronic-Implementation of Maternal Death/Mortality Review, *MDR* Maternal death review, *TFA* Theoretical Framework of Acceptability

Affective attitude (reviewers’ feelings towards E-MPATHY). *Perceived the need of re-learning and updating reviewers’ knowledge.* All the reviewers perceived a positive feeling and appreciation due to the acquired personal benefits to use the E-MPATHY. They felt the motivation to relearn and update the required scientific evidence to revise their own clinical practice.

“I am glad to update my clinical knowledge [while using this tool] in the same time.” (A distric reviewer, 46 years old).

Burden (perceived amount of effort required in using E-MPATHY). *Demanding commitment to provide extra time to learn the updated guidelines.* The reviewers expressed concern that the review model encouraged them to continuously learn the new contents of the maternal and child health handbook.

“Using this tool, it forced me to learn the content of the updated MCH [Maternal and Child Health] handbook. I feel I have to read it every single day.” (A provincial reviewer, 40 years old).

Ethicality (the extent in which the E-MPATHY was perceived as having a good fit with an individual value system). *Self-reflection after being able to identify the third delay.* Interestingly, in term of the ethicality construct, some reviewers reported that their experiences in using this tool promoted self-reflection to improve their awareness of the possible outcome of delays in the clinical practice setting. One reviewer shared the following insight:

“[my experience with E-MPATHY] reviewing cases makes me aware of my own practices. I intended to delay cesarean section for my patient with severe preeclampsia until the folowing morning [but] I decided to do the emergency cesarean section considering the high risk of death in such a case.” (A district reviewer, 42 years old).

Intervention coherence (understanding of how using E-MPATHY works in formulating evidence-based recommendation). *The reviewers constantly use clinical guidelines when disagreement occurs*. This tool serves as a practical clinical standard which is particularly useful when there are reviewers’ different ways of clinical management to reach consensus in assigning the cause of maternal death and its contributing factors. This was clearly articulated by one of the reviewers:

[to solve the disagreement] “let’s look it up in the clinical guideline”. (A provincial reviewer, 40 years old).

Opportunity costs (whether using the E-MPATHY would interfere with other important priorities in formulating evidence-based recommendation). *Using E-MPATHY for reviews guided more appropriate alternative recommendations.* Some reviewers felt offering the evidence-based recommendation was a priority. All the reviewers perceived a positive learning about the advantages of E-MPATHY. The reviewers expressed their perceived benefits of the E-MPATHY, such as shorter review time, accuracy, lower risk of misclassification, and opportunity of updating their knowledge to the latest evidence or guidelines. They highlighted the familiarity of doing reviews using clinical standards and evidence-based guidelines to reveal maternal care deficiencies and proposed suitable recommendations.

“Using this tool helps me to choose the right recommendation.” (A district reviewer, 46 years old)

Perceived effectiveness (perceived accurate identification delays and recommendation). *Mixed perceptions whether the tool was effective to identify delays and formulate recommendation between district and provincial teams*. Reviewers from different levels expressed that the effectiveness the E-MPATHY tool would be unique to each case, including COVID-19, triple elimination, and task-shifting. Regarding COVID-19, the main concern of the district team was the tool’s potential ineffectiveness due to frequent updates in guidelines for specific cases such as patients with COVID-19.

“If we use clinical updated guideline [E-MPATHY], it would consistently conclude with failure to meet standard of care despite the fact that the health professionals have complied to the older standard.” (A district reviewer, 42 years old).

On the other hand, higher level of reviewers felt that E-MPATHY would be effective for all COVID-19 cases because of their intention to identify any deficiency of health care services.

“It is relevant to use E-MPATHY for the maternal death due to COVID-19 occurred before the latest guideline is valid because the goal of maternal audit is to identify the deficiency in the health care.” (A provincial reviewer, 52 years old).

Task-shifting to midwives of selected interventions also interfered with the perceived effectiveness of the tool. This present study found that the lowest agreement was found in the delay of the intrapartum period related to professional birth attendants. While the provincial reviewers perceived the task shifting as a delay, the district reviewers considered it as acceptable considering the limited number of attending OB/GYN professionals. Concerning the inadequate triple elimination, the district team did not perceive the cases with inadequate testing as contributing factors to maternal death. Conversely, the provincial team associated this inadequacy as one of the deficiencies in maternal care.

Self-efficacy (perceived confidence in using E-MPATHY). *The reviewers were able to review more cases per day routinely.* All reviewers acknowledged the applicability of the tool indicating that they felt confidence in using the tool. The reviewers showed an increasing confidence indicated by more number of reviewed cases week by week. A reviewer would take responsibilities to review more than the assigned number of cases.

“[I thought that I would review more than] two cases?” *(A district reviewer, 46 years old).*

## Discussion

To our knowledge, this study represents the first attempt to develop a maternal death review decision-support tool designed to foster a supportive learning environment within review processes. The findings demonstrate that E-MPATHY is valid, reliable, and acceptable for identifying underlying causes and delay patterns of maternal deaths, as well as for generating evidence-based recommendations across pregnancy periods at the district level.

Our previous study highlighted the need for a structured framework and tool to strengthen maternal audit and review processes in decentralized health systems [16]. The E-MPATHY was designed to facilitate translating scientific evidence by assigning the content of change in MDR to support faster policy change applying the priciple of ‘local solutions to local problems’ [13, 18]. 

To elicit the pattern of maternal death and design an appropiate strategies for quality improvement, the E-MPATHY, developed with a robust methodology based on the ‘three delays’ model, a familiar program at the grassroots level [24], in each pregnancy period was proposed. However, the original model has limitations, particularly its focus on identifying delays after complications occur and its limited support for preventive and action-oriented strategies [25]. To address these gaps, the model was adapted and integrated with evidence-based guidelines to enable the identification of contributing factors across all pregnancy periods and to support the formulation of recommendations for primary (preventing disease before it occurs), secondary (detecting and treating early) and tertiary (preventing further complications of an advanced disease) prevention [29].

 In several high-income countries, such as the United Kingdom, maternal death reviews are conducted through confidential enquiry systems that rely on multidisciplinary expert panels and comprehensive clinical documentation [30, 31], reflecting the importance of multidisciplinary expertise in preventing misclassification and improving diagnostic accuracy [32]. While these systems enable in-depth analyses of maternal deaths, their implementation requires substantial resources and specialized expertise [30, 31]. In contrast, maternal death review processes in many low- and middle-income countries are typically conducted at the district or facility level, where tool, resources, data completeness, and access to multidisciplinary specialists may be limited. These constrains often affect the consistency and depth of case analysis, including the accurate identification of causes of death and contributing delays [10, 33, 34].

To address these gaps, the E-MPATHY offers a structured and feasible approach to strengthening maternal death review processes in resource-limited settings. The E-MPATHY decision-support tool was designed to address these contextual challenges by providing a structured analytical framework that integrates clinical evidence, standardized ICD-MM classification, and systematic identification of delay patterns across pregnancy periods. By guiding users through a consistent review process and improving the completeness and quality of data collection, it enhances the reliability of maternal death reviews. This is reflected in the almost perfect inter-rater agreement in assigning underlying causes of death between district- and provincial-level reviewers, even in the absence of multidisciplinary specialists. This high level of agreement may be attributed to the integration of clinical evidence and standardized classification, which reduces ambiguity and limits subjective interpretation [10, 35, 36]. This, in turn, supports more consistent and evidence-based decision-making across review levels.

In addition, this level of agreement is further supported by the qualitative assessment of acceptability [28]. Positive perceptions across several TFA constructs, including affective attitude, burden, intervention coherence, opportunity costs, perceived effectiveness, and self-efficacy, indicate that the tool was perceived as understandable, feasible, and supportive in generating evidence-based recommendations. Our results indicate that E-MPATHY not only improves technical aspects of review process but also strengthens reviewers’ confidence and engagement.

Despite these strengths, some discrepancies were observed in identifying contributing factors related to delays and in formulating recommendations. These aspects appeared to be more challenging than assigning the underlying cause of death. One possible explanation for these discrepancies is that identifying contributing factors and developing recommendations require greater interpretative judgment and are more influenced by contextual health system factors, including variations in service organization, resource availability, referral systems, and task-shifting practices [15, 37]. This aligns with previous evidence indicating that the development of evidence-informed interventions is shaped by multiple layers of decision-making, including individual, social, and health system considerations [15, 38]. Such variations have also been reported in other settings. For example, differences in assigning maternal causes of death between the United Kingdom Confidential Enquiry into Maternal Deaths and the Dutch Audit Committee on Maternal Mortality and Morbidity have been attributed to contextual differences, including concerns about the magnitude and classification of obstetric morbidity [39].

Similarly, these findings highlight the importance of contextual factors in shaping how cases are interpreted within maternal death reviews. This may reflect the unique characteristics of each case under review, including specific contextual factors such as task-shifting practices. Although the task-shifting is widely recognized as an approach to address gaps in service provision [37, 40, 41], cleared criteria for task-shifting decisions may be required in situations involving delays. These criteria may promote more consistent assessments and improve the quality of care, particularly in relation to the third delay.

These findings have important implications for policy and practice. At the policy level, integrating the E-MPATHY decision-support tool into national MDSR guidelines may support more standardized and evidence-based maternal death review processes. At the practice level, the tool facilitates more consistent case analysis and strengthen reviewer’s capacity to degenerate context-spesific recommendations. By promoting structured, evidence-informed learning grounded in clinical standards and guidelines, it also enhances acceptability and supports greater awareness among reviewers in routine practice*.*

This, in turn, enables reviewers to take on a more active role as “champions” of quality improvement, rather than reinforcing blame-oriented approaches or contributing to a culture of silence around medical errors. As described by Pattinson and Berg, maternal review team consists of clinicians responsible for providing care, where “champion” doers play a key role in identifying causes of death using evidence-based knowledge and formulating policy relevant recommendations. Reviewers who are actively engaged are more likely to conduct constructive and accountable reviews [15, 18].

By fostering such engagement, the DST may contribute to creating a supportive learning environment and strengthening reviewers’ awareness as a foundation for behavior-change. This is essential for sustaining the functional cycle of maternal death surveillance and response, where review teams not only generate an evidence-based recommendation but also promote continuous learning and improvement in the quality of care [18, 42].

Such a structured review process is particularly relevant in Indonesia, where an increasing proportion of maternal deaths occur in referral hospitals [43]. By integrating standardized ICD-MM classification, delay analysis across pregnancy periods, and evidence-based clinical standards, the structured review process facilitated by E-MPATHY enables reviewers to examine contributing factors before referral, during transfer, and after arrival at referral facilities. These structured review findings may also contribute to the response component of MDSR by supporting the development of context-specific, evidence-based recommendations that can inform district-level quality improvement within Indonesia's decentralized health system.

### Strengths and limitations

There are several notable strengths in this study. The application of E-MPATHY was comprehensively developed by involving the representatives of the different regions (western, central, and eastern areas in Indonesia) through needs assessment and validation. The online reviews during the study using the Zoom platform, which modeled real review setting, demonstrated the electronic tool's display, readability, and usability. The rigorous need assessment found the importance of work performance using the tool indicated by reliability and acceptability to prevent the common workload as an unintended consequence of digital tools [44].

Some limitations of this study should be considered. First, as the acceptability was based on the self-reporting during observation, response bias cannot be avoided. Second, the reliability assignment involved reviewers and cases from only one region in Indonesia, which may limit the generalizability of the findings to other regions with different contextual characteristics. Given these limitations, further research is needed to examine the tool’s performance across diverse regions in Indonesia, taking into account regional disparities. Future studies should also explore how multidisiplinary team dynamics in maternal death reviews, including district health officials and facility-based professionals, to better understand how individual, societal, and health system factors shape the formulation of evidence-based recommendations using the DST. Additional research is needed to assess the implementation of the DST, including its adoption, feasibility, and sustainability in different health system contexts, as weel as its potential for scale-up in other countries.

## Conclusions

The E-MPATHY shifts maternal death review from conventional descriptive audit approaches toward a more structured and action-oriented process. It offers a valuable tool for generating context-specific, evidence-based recommendations and strengthening the learning process within MDSR, particularly at the district level in resource-limited settings. With further refinement and validation across diverse regions, and consideration of task-shifting within policy framework, E-MPATHY has the potential to be integrated into national MDSR guidelines to support more standardized and scalable quality improvement efforts. To support such integration, effective large-scale implementation would require embedding the tool within existing review workflows, providing training for reviewers, and ensuring alignment with national clinical guidelines and health information systems.

## Supplementary Information

Supplementary Material 1.

Supplementary Material 2.

Supplementary Material 3.

## Data Availability

The datasets generated and/or analyzed during the current study are not publicly available due to the confidentiality nature of the participants and maternal death cases but are available from the corresponding author on reasonable request.

## Abbreviations

E-MPATHY: Electronic-Implementation of Maternal Death/Mortalit**y** Review
DST: Decision-Support Tool
MMR: Maternal mortality ratio
MDA: Maternal death audit
WHO: World Health Organization
MDSR: Maternal death surveillance and response
MDR: Maternal death review
CEMD: Confidential Enquiry into Maternal Deaths
TFA: Theoretical Framework of Acceptability
ICD-MM: International Classification of Diseases-Maternal Mortality
MDGs: Millenium Development Goals
POGI: *Perkumpulan Obstetri dan Ginekologi* *Indonesia* (Indonesian Society of Obstetrics and Gynecology)
IBI: *Ikatan Bidan Indonesia* (Indonesian Midwives Association)
IDI: *Ikatan Dokter Indonesia* (Indonesian Medical Association)
FGDs: Focus group discussions
DHO: District health office
