# Supplementary material for: E-MPATHY: Development, validation, reliability, and acceptability of a decision-support tool for district-level maternal death review in Indonesia
Source: BMC Pregnancy Childbirth. 2026 Jul 30;26:1219. doi: 10.1186/s12884-026-09735-5 (PMC13711916; doi:10.1186/s12884-026-09735-5)
Supplement: Supplementary file 1 [file 12884_2026_9735_MOESM1_ESM.pdf]

Appendix 1. Concept/Need, theme, and category of MDR tool in districts/cities

Concept/need	Sub-category	Category	Theme
Cause of death analysis	Improved data collection	1. Changing the name of the audit to encourage quality improvements	1. Framework
	Improved capacity-building processes		
	Maternal audit goals	2. Basic comprehension of audit	
	Benefits of the maternal audit		
	Principles of the maternal audit		
	Roles and authority of the MDA team		
	The legal aspect of MDA		
	The audit phases	3. Comprehension of maternal audit process/cycle	
	Workflow and deadline of the audit phases		
	A concise guideline on maternal audit phases		
	Periodic training for the audit team	4. Skills and competency of the maternal audit team	
	Implementation of Obstetricians and Gynecologists as leaders in the maternal audit		
	Guidelines facilitating the effects of maternal audit		
	Healthcare facilities' reports on chronology and medical audit results	5. Follow-up reporting	
	Setting the deadline for maternal death follow-up reports		
	Following up the audit results from the district health office to healthcare facilities		
	The application is effective and efficient from data collection to reviewing data and generating recommendations	6. Convenient quality tools/application systems	
	The application presents the maternal audit cycle progress on the dashboard		
	The application can maintain data confidentiality.		
	The application facilitates data for cross-border cases.		
	The application stores databases for monitoring the MDSR action cycle		
	Comprehensive closed review that supports the MDA's principles	7. Organizing the initial step of MDR	

Concept/need	Sub-category	Category	Theme
	Understanding how to organize the review and deliver recommendations for cross-border case		
	Understanding maternal death definition, causes, and criteria	8. Integrated knowledge of maternal death	2. Maternal death identification
	Comprehension of cross-border cases	9. Cross-border case	
	Supervision on data completeness for review	10. Data completeness on the Dashboard	3. Systematic analysis
	Supervision on maternal review phases		
	Presented in a familiar format, such as a case report on PowerPoint	11. Case chronology	
	ANC data completeness, early pregnancy care, intrapartum, postpartum, referral, and followed with timely case chronology		
	Synchronized chronology between verbal autopsy and the form of maternal death in facilities		
	No overlapping stories in the maternal death form in each facility for referral cases		
	The form describes the case development in points of the concept		
	Information about facility-based MDR and its recommendations		
	Verification of maternal death form by health facilities management/doctors in charge before submitted to DHO		
	Divided into components of antenatal, early pregnancy, ante-intrapartum and postpartum management	12. Review guidelines	
	The antenatal care component highlights early detection and referral.		
	Early pregnancy care and the antepartum-intrapartum component underline supervision, labor monitoring, emergencies, and referral management.		
	The postpartum component highlights supervision, emergencies, and referral management.		
	Based on current evidence and best practice	13. Review standards	

Concept/need	Sub-category	Category	Theme
	Using three delays model framework		
	Clinical guidelines are used as references for the review		
	The review in management uses scientific evidence as the standard		
	Concise, unlike a book		
	Management of antenatal, early pregnancy, antepartum-intrapartum, and postpartum	14. Review summary	
	Assigning the cause of maternal death		
	Identifying delays in maternal death		
	Identifying medical and non-medical factors that contribute to maternal death		
	Guidelines for a review summary		
Recommendations	Based on evidence and best practices	15. Recommendation guidelines	4. Practical recommendations
	Based on clinical management guidelines and intervention packages for management and community aspects		
	Using the 'three delays' model framework	16. Recommendation models	
	Appointing the person in charge and the deadline for action		
	The programs must be followed up immediately by DHO	17. Short-term program	5. Follow-up plans
	Implementation of the maternal audit action cycle as integrated supervision and evaluation of the Maternal and Child Health Program	18. Long-term program	

DHO, district health office; MDA, maternal death audit; MDR, maternal death review; MDSR, maternal death surveillance, and response.
