# Supplementary material for: E-MPATHY: Development, validation, reliability, and acceptability of a decision-support tool for district-level maternal death review in Indonesia
Source: BMC Pregnancy Childbirth. 2026 Jul 30;26:1219. doi: 10.1186/s12884-026-09735-5 (PMC13711916; doi:10.1186/s12884-026-09735-5)
Supplement: Supplementary file 2 [file 12884_2026_9735_MOESM2_ESM.pdf]

Appendix 2. Panelists' Likert-Scale Scores

POINTS OF INTEREST	SA n (%)	A n (%)	N n (%)	D n (%)	SD n (%)	Total
**A Cause of death analysis**						
1 Framework						
a Changing the name of the audit E-MPATHY to encourage quality improvements						
Improved data collection	7 (100)	0	0	0	0	35
Improved capacity-building processes	7 (100)	0	0	0	0	35
b Basic comprehension of audit						
Maternal audit goals	7 (100)	0	0	0	0	35
Benefits of maternal audit	7 (100)	0	0	0	0	35
Principles of maternal audit	6 (85.7)	1 (14.3)	0	0	0	34
Roles and authority of the MDA teams	5 (71.4)	2 (28.6)	0	0	0	33
The legal aspects of MDA	5 (71.4)	1 (14.3)	1 (14.3)	0	0	32
c Comprehension of maternal audit process/cycle						
Audit phases	5 (71.4)	2 (28.6)	0	0	0	33
Workflow and deadline of the audit phases	6 (85.7)	1 (14.3)	0	0	0	34
Concise guidelines on maternal audit phases	7 (100)	0	0	0	0	35
d Skills and competency of the maternal audit team						
Periodic training for the audit team	5 (71.4)	1 (14.3)	1 (14.3)	0	0	32
Implementation of Obstetricians and Gynecologists as the leaders in maternal audit	6 (85.7)	1 (14.3)	0	0	0	34
Guidelines facilitating the effect of maternal audit	7 (100)	0	0	0	0	35
e Follow-up reporting						
Healthcare facilities' reporting on chronology and medical audit results	7 (100)	0	0	0	0	35
Deadline setting for maternal death follow-up reports	5 (71.4)	2 (28.6)	0	0	0	33
Follow-up of audit results from DHO to healthcare facilities	7 (100)	0	0	0	0	35

POINTS OF INTEREST			SA n (%)	A n (%)	N n (%)	D n (%)	SD n (%)	Total
	f	Convenient and quality tools/application systems						
		The application is effective and efficient from data collection to reviewing data and generating a recommendations	7 (100)	0	0	0	0	35
		The application presents the maternal audit cycle progress on the dashboard	6 (85.7)	1 (14.3)	0	0	0	34
		The application can maintain data confidentiality	7 (100)	0	0	0	0	35
		The application facilitates data for cross-border cases	6 (85.7)	1 (14.3)	0	0	0	34
		The application stores databases for monitoring the MDSR action cycle	6 (85.7)	1 (14.3)	0	0	0	34
	g	Organizing early action						
		Comprehension of closed review supporting the principles of MDA	6 (85.7)	1 (14.3)	0	0	0	34
		Understanding how to organize the review and deliver recommendations for cross-border cases	6 (85.7)	1 (14.3)	0	0	0	34
**2**	**Maternal death identification**							
	a	Integrated knowledge of maternal death						
		Understanding the maternal death definition, causes, and criteria	7 (100)	0	0	0	0	35
	b	Cross-border cases						
		Comprehension of cross-border cases	7 (100)	0	0	0	0	35
**3**	**Systematic Analysis**							
	a	Data completeness on the dashboard						
		Supervision on data completeness for the review	7 (100)	0	0	0	0	35
		Supervision on maternal review phases	7 (100)	0	0	0	0	35
	b	Case chronology						
		Presented in a familiar format, such as a case report on Powerpoint	6 (85.7)	1 (14.3)	0	0	0	34
		ANC data completeness, early pregnancy care, intrapartum, postpartum, referral, and followed with timely case chronology	7 (100)	0	0	0	0	35

POINTS OF INTEREST	SA n (%)	A n (%)	N n (%)	D n (%)	SD n (%)	Total
Synchronized chronology between verbal autopsy and the form of maternal death in facilities	6 (85.7)	1 (14.3)	0	0	0	34
No overlapping stories in the maternal death form in each facility for the referral cases	6 (85.7)	1 (14.3)	0	0	0	34
The form describes the case development in concise points	6 (85.7)	1 (14.3)	0	0	0	34
Information on facility-based MDR and its recommendations	6 (85.7)	1 (14.3)	0	0	0	34
Verification of maternal death form by health facilities management/doctors in charge before submitting to DHO	7 (100)	0	0	0	0	35
c Review Guidelines						
Divided into components of antenatal management, early pregnancy, ante-intrapartum, and postpartum management	6 (85.7)	1 (14.3)	0	0	0	34
The antenatal component highlights early detection and referral	6 (85.7)	1 (14.3)	0	0	0	34
The early detection during the antenatal period highlights the quality of ANC and the implementation of birth preparedness and complications readiness	5 (71.4)	2 (28.6)	0	0	0	33
Monitoring during early pregnancy and antepartum focuses on patient care and early detection of emergencies	6 (85.7)	1 (14.3)	0	0	0	34
The referral assessment in early pregnancy and antepartum components pointed out the aspects of stabilization, communication, and transportation for emergency referral and staff accompaniment for planned referral	6 (85.7)	1 (14.3)	0	0	0	34
Intrapartum component points out labor progression, emergencies, and referral	5 (71.4)	2 (28.6)	0	0	0	33
Labor progression, pointed out in the intrapartum component, focuses on monitoring during labor	5 (71.4)	2 (28.6)	0	0	0	33
Emergency management in intrapartum focuses on monitoring, stabilization, and obstetric intervention	5 (71.4)	2 (28.6)	0	0	0	33
Referral care in intrapartum focusing on stabilization, communication, and transportation	6 (85.7)	1 (14.3)	0	0	0	34

POINTS OF INTEREST	SA n (%)	A n (%)	N n (%)	D n (%)	SD n (%)	Total
The postpartum component highlights the monitoring, emergencies, and referral	5 (71.4)	2 (28.6)	0	0	0	33
The postpartum component points out monitoring during in-patient and outpatient care at healthcare facilities as well as the quantity and quality of a postpartum visit	6 (85.7)	1 (14.3)	0	0	0	34
Emergency management in postpartum focuses on monitoring, stabilization, and obstetric intervention	5 (71.4)	2 (28.6)	0	0	0	33
Referral care in postpartum focuses on stabilization, communication, and transportation for emergency referral and staff accompaniment for planned referral	6 (85.7)	1 (14.3)	0	0	0	34
d Review standard						
Based on current best evidence and best practices	6 (85.7)	1 (14.3)	0	0	0	34
Using a three-delays model framework	6 (85.7)	1 (14.3)	0	0	0	34
Clinical guidelines are used as references for review	5 (71.4)	2 (28.6)	0	0	0	33
The review in the management uses scientific evidence as the standard	5 (71.4)	2 (28.6)	0	0	0	33
e Review conclusion						
Management of antenatal, early pregnancy, ante-intrapartum, and postpartum	6 (85.7)	1 (14.3)	0	0	0	34
Assigning the cause of maternal death	6 (85.7)	1 (14.3)	0	0	0	34
Causes of death are based on the ICD-MM classification	5 (71.4)	2 (28.6)	0	0	0	33
The medical factors contributing to maternal death are based on ICD-10	6 (85.7)	1 (14.3)	0	0	0	34
The medical and non-medical factors contributing to maternal death are aligned with the three-delay model framework	7 (100)	0	0	0	0	35
**B Recommendations**						
**1** Effective recommendations						
a Recommendation guidelines						
Based on evidence and best practices	5 (71.4)	2 (28.6)	0	0	0	33
Based on clinical management guidelines and intervention packages for management and community aspects	3 (42.9)	4 (57.1)	0	0	0	31

POINTS OF INTEREST	SA n (%)	A n (%)	N n (%)	D n (%)	SD n (%)	Total
b Recommendation models						
Using the 'three delays' model framework	5 (71.4)	2 (28.6)	0	0	0	33
Determining the person in charge and the deadline for actions	4 (57.1)	3 (42.9)	0	0	0	32
**2 Follow-up program**						
a Short-term programs						
The programs must be followed up immediately by DHO	6 (85.7)	1 (14.3)	0	0	0	34
b Long-term programs						
The maternal audit action cycle in the form of integrated supervision and evaluation of the Maternal and Child Health Program	5 (71.4)	2 (28.6)	0	0	0	33

Notes: Ratings were calculated based on the number of panelists choosing the Likert scale multiplied by the conversion of scale value (Strongly Agree = 5; Agree = 4; Neutral = 3; Disagree = 2; and Strongly Disagree = 1; ANC, antenatal care; DHO, district health office; ICD-MM, International Classification of Diseases – Maternal Mortality; ICD-10, International Classification of Diseases and Related Health Problems - 10th Revision; E-MPATHY, **E**lectronic-**Imp**lementation of M**a**ternal Dea**th**/Mortalit**y** Review; MDR, maternal death review.
