# Supplementary material for: E-MPATHY: Development, validation, reliability, and acceptability of a decision-support tool for district-level maternal death review in Indonesia
Source: BMC Pregnancy Childbirth. 2026 Jul 30;26:1219. doi: 10.1186/s12884-026-09735-5 (PMC13711916; doi:10.1186/s12884-026-09735-5)
Supplement: Supplementary file 3 [file 12884_2026_9735_MOESM3_ESM.pdf]

Appendix 3. Developing the E-MPATHY website based on needs assessment findings

Themes	Component	Navigation bar	User Components
**I. Cause of death analysis aspect**			
Framework	The naming of the audit into E-MPATHY	Navigation menu	DHO, reviewer, healthcare facilities
	Basic comprehension of the audit process	Guideline- FAQ E-MPATHY	
	Skills and competency of the maternal audit team	Guideline - FAQ	
	Follow-up reporting procedures	Guideline - FAQ, message, recommendation implementation	
	Convenient and high-quality tool/application systems	Web E-MPATHY	
	Organizing the initial steps of MDR	Guideline - FAQ	
Maternal death identification	Integrated knowledge of maternal death	Guideline - FAQ, dashboard	DHO
		Guideline- FAQ	Reviewer
		Guideline – FAQ, death notification form, maternal verbal autopsy form, ANC, Early pregnancy, ante-intrapartum, postpartum forms for maternal death in facilities, facility-based MDR	Related healthcare facilities
	Cross-border cases	Guideline - FAQ, dashboard	DHO, reviewer, healthcare facilities
Systematic analysis	Data completeness on the dashboard	Dashboard	DHO
	Case chronology	Antenatal, early pregnancy, ante-intrapartum, and postpartum forms for maternal death in facilities, facility-based MDR	Healthcare facilities that provide care to the cases.
		Review	DHO, reviewer
	Review guidelines	Review	DHO, reviewer
	Review standards	Review	
	Review summary	Review	
**II. Recommendation Aspect**			
Practical recommendations	Recommendation guidelines	Review summary	DHO, reviewer
	Recommendation models	Review summary	
Follow-up plans	Long-term program	Review summary	DHO, reviewer
	Short-term program	Review summary	

ANC, antenatal care; DHO, district health office; E-MPATHY, **E**lectronic-I**mp**lementation of M**a**ternal Dea**th**/Mortalit**y** Review; FAQ, frequently asked questions; MDR, maternal death review.
